# Supplementary material for: A Novel Hypothalamic Factor, Neurosecretory Protein GM, Causes Fat Deposition in Chicks
Source: Front Physiol. 2021 Oct 25;12:747473. doi: 10.3389/fphys.2021.747473 (PMC8573243; doi:10.3389/fphys.2021.747473)
Supplement: Supplementary file 1 [file Data_Sheet_1.PDF]

Supplementary Material

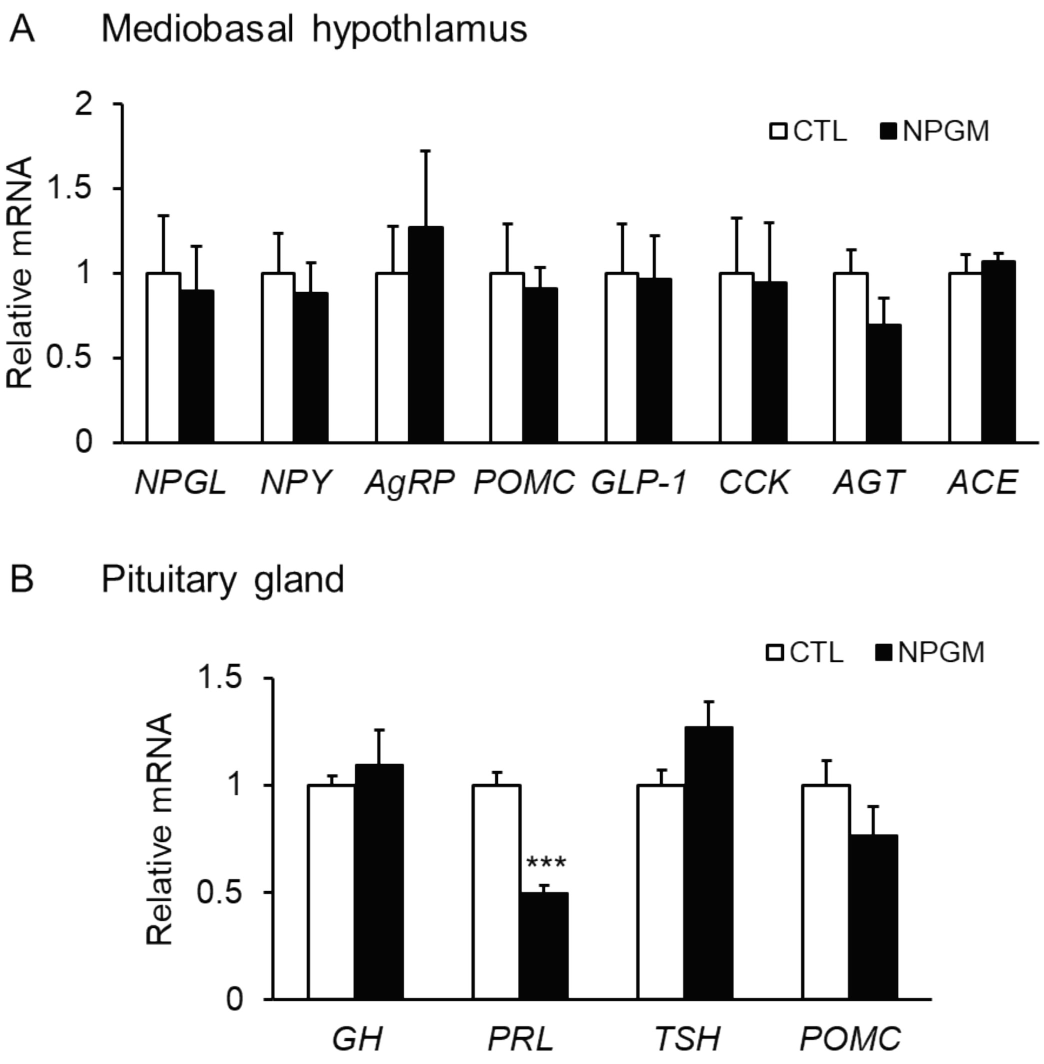

Supplementary Figure 1. Effect of chronic i.c.v. infusion of NPGM on the mRNA expression of neurosecretory protein GL (*NPGL*), neuropeptide Y (*NPY*), agouti-related peptide (*AGRP*), pro-opiomelanocortin (*POMC*), glucagon-like peptide-1 (*GLP-1*), cholecystokinin (*CCK*), angiotensinogen (*AGT*), and angiotensin-converting enzyme (*ACE*) in the hypothalamus (A). In the pituitary gland, we chose growth hormone (*GH*), prolactin (*PRL*), thyroid-stimulating hormone (*TSH*), and *POMC* (B). The results were obtained 2 weeks after infusion of the vehicle (control; CTL) and NPGM. Data are expressed as the mean ± SEM (n = 7–8). Data were analyzed by Student's *t*-test. An asterisk indicates a statistically significant difference (****P* < 0.005).

Supplementary Table 1. Sequences of oligonucleotide primers for real-time PCR

Gene	Forward primer	Reverse primer	Accession no.
ACC	AATGGCAGCTTTGGAGGTGT	TCTGTTTGGGTGGGAGGTG	NM_205505.1
FAS	CCAACGATTACCCGTCTCAA	CAGGCTCTGTATGCTGTCCAA	NM_205155.2
SCD1	AGTGGTGTTGCTGTGCTTCA	CTAAGGTGTAGCGCAGGATG	NM_204890.1
ME	AGTGCCTACCTGTGATGTTG	GGCTTGACCTCTGATTCTCT	NM_204303.1
PPARγ	TCAAGCATTTCTTCACCACACT	ATTGCACTTTGGCAATCCTGG	NM_001001460.1
FATP1	TACAATGTGCTCCAGAAGGG	GTCTGGTTGAGGATGTGACTC	NM_001039602.2
PPARα	TGCTGTGGAGATCGTCCTGGT	AGAGGAAGATATCGTCAGGATGG	NM_001001464.1
CPT1a	TGATCTGAAGAAGAACCCTGAGAT	TCCAAAGCGATGAGAATCCG	NM_001012898.1
LPL	CAGTGCAACTTCAACCATACCA	AACCAGCCAGTCCACAACAA	NM_205282.1
ATGL	CACTGCCATGATGGTCCCCTA	CCACAAGGAGATGCTGAAGAA	NM_001113291.1
CGI-58	ACCGTGGTTTATGGAGCACG	GAAACAGTGTGCAAACAGAGCC	NM_001278145.1
NPGL	CTAGGAAAAAGACAGCTTGC	CTTTCTTCGTCAGAACTGGT	NM_001389496
NPY	ACATGGCCAGATACTACTCG	ACAAGAGGTCTGAGATCAGTG	NM_205473.1
AgRP	AGGCCAGACTTGGATCAGATG	ACTCCAGGAGGCGGACAC	NM_001031457.1
POMC	AGGAGTCGGCTGAGAGTTA	TTCCTCTTCCTCCTCTTCTT	NM_001031098.1
GLP-1	CGTCATTCACAAGGCACATTC	GTCATTCTCTTTGTCCTCCTGTCC	NM_205260.5
CCK	AGGTTCCACTGGGAGGTTCT	CGCCTGCTGTTCTTTAGGAG	NM_001001741.1
AGT	AGCAGGTTTGAGAGGCAATGA	GATTCCACCACTTCCCCAGG	XM_419584.7
ACE	GCCAAACTCAGGGAGGTGTT	CCCCAGCGTCCATCTTATCC	NM_001167732.1
GH	CCAGAGTCCATCACAATACC	AGCCAACAGAGAGAAGATGA	NM_204359.2
PRL	AAGAAGCTCCAGATACCATTCTCT	GAGAGTAAATTTCATTTCCAGCAT	NM_205466.2
TSH	CCACCATCTGCGCTGGAT	GCCCGGAATCAGTGCTGTT	NM_205063.3
ACTB	CCAGAGTCCATCACAATACC	AGCCAACAGAGAGAAGATGA	NM_205518.1
